# Factors associated with elevated SARS-CoV-2 immune response in children and adolescents

**DOI:** 10.3389/fped.2024.1393321

**Published:** 2024-08-15

**Authors:** Sarah E. Messiah, Rhiana Abbas, Emma Bergqvist, Harold W. Kohl, Michael D. Swartz, Yashar Talebi, Rachit Sabharwal, Haoting Han, Melissa A. Valerio-Shewmaker, Stacia M. DeSantis, Ashraf Yaseen, Henal A. Gandhi, Ximena Flandes Amavisca, Jessica A. Ross, Lindsay N. Padilla, Michael O. Gonzalez, Leqing Wu, Mark A. Silberman, David Lakey, Jennifer A. Shuford, Stephen J. Pont, Eric Boerwinkle

**Affiliations:** ^1^ Department of Epidemiology, UTHealth Houston School of Public Health, Dallas, TX, United States; ^2^ Center for Pediatric Population Health, UTHealth Houston School of Public Health, Dallas, TX, United States; ^3^ Department of Pediatrics, McGovern Medical School at UTHealth Houston, Houston, TX, United States; ^4^ Department of Biostatistics and Data Science, UTHealth Houston School of Public Health, Houston, TX, United States; ^5^ Department of Epidemiology, UTHealth Houston School of Public Health, Austin, TX, United States; ^6^ Department of Kinesiology and Health Education, The University of Texas at Austin, Austin, TX, United States; ^7^ Department of Health Promotion and Behavioral Sciences, UTHealth Houston School of Public Health, Brownsville, TX, United States; ^8^ Department of Epidemiology, UTHealth Houston School of Public Health, Houston, TX, United States; ^9^ Clinical Pathology Laboratories, Austin, TX, United States; ^10^ The University of Texas System, Austin, TX, United States; ^11^ The University of Texas at Tyler Health Science Center, Tyler, TX, United States; ^12^ Texas Department of State Health Services, Austin, TX, United States

**Keywords:** SARS-CoV-2, children, adolescents, immune response, epidemiology

## Abstract

**Background:**

Understanding the distinct immunologic responses to SARS-CoV-2 infection among pediatric populations is pivotal in navigating the COVID-19 pandemic and informing future public health strategies. This study aimed to identify factors associated with heightened antibody responses in children and adolescents to identify potential unique immune dynamics in this population.

**Methods:**

Data collected between July and December 2023 from the Texas Coronavirus Antibody REsponse Survey (Texas CARES), a statewide prospective population-based antibody survey among 1-to-19-year-old participants, were analyzed. Each participant had the following data available for analysis: (1) Roche Elecsys® Anti-SARS-CoV-2 Immunoassay for Nucleocapsid protein antibodies (Roche N-test), (2) qualitative and semi-quantitative detection of antibodies to the SARS CoV-2 spike protein receptor binding domain (Roche S-test), and (3) self-reported antigen/PCR COVID-19 test results, vaccination, and health status. Statistical analysis identified associations between participant characteristics and spike antibody quartile group.

**Results:**

The analytical sample consisted of 411 participants (mean age 12.2 years, 50.6% female). Spike antibody values ranged from a low of 6.3 U/ml in the lowest quartile to a maximum of 203,132.0 U/ml in the highest quartile in the aggregate sample. Older age at test date (OR = 1.22, 95% CI: 1.12, 1.35, *p* < .001) and vaccination status (primary series/partially vaccinated, one or multiple boosters) showed significantly higher odds of being in the highest spike antibody quartile compared to younger age and unvaccinated status. Conversely, fewer days since the last immunity challenge showed decreased odds (OR = 0.98, 95% CI: 0.96, 0.99, *p* = 0.002) of being in the highest spike antibody quartile vs. more days since last immunity challenge. Additionally, one out of every three COVID-19 infections were asymptomatic.

**Conclusions:**

Older age, duration since the last immunity challenge (vaccine or infection), and vaccination status were associated with heightened spike antibody responses, highlighting the nuanced immune dynamics in the pediatric population. A significant proportion of children/adolescents continue to have asymptomatic infection, which has important public health implications.

## Introduction

Identifying distinct immunologic responses among children with SARS-CoV-2 infection is key to understanding multiple nuances of the COVID-19 pandemic. Pediatric patients are generally less symptomatic compared to adults, yet studies consistently show robust, long-lasting antibody responses to both infection and vaccination via nucleocapsid (N) and/or spike (S) antibodies, respectively, regardless of symptom or disease severity, lasting at least one year (1–3). Although some studies of adults have shown subsets of individuals who mount very high immune responses to vaccination or infection (4), or no response at all (5), this has not been widely investigated in children or adolescents. Understanding factors driving the variation in immune reactions in children is crucial for advancing pediatric immunology and shaping effective public health strategies as the pandemic to endemic transition continues (6).

SARS-CoV-2 infections in children typically lead to mild or asymptomatic cases, supported by studies indicating rare occurrences of severe acute infections, multisystem inflammatory syndrome in children (MIS-C), and long COVID (7–9). Current evidence points to various factors, such as innate immunity and local tissue responses, that are associated with a lower risk of severe disease in children (7, 8). Research has shown consistent, strong antibody responses in children, especially among those with hybrid immunity (acquired through both natural infection and vaccination) irrespective of symptom severity, symptomatic infection, age, sex, or body mass index (BMI) (10–15). Questions remain about what factors are associated with high to very high, or conversely, no immune response. Adult studies have shown younger age, female sex (6, 16), more vaccinations/boosters, fewer days since vaccination, absence of hypertension (6), and experiencing a breakthrough infection (17) are associated with higher immune response. Conversely, the presence of autoimmune disorders, diabetes and hypertension (18), kidney disease, being a smoker (19), being a transplant recipient (6), and race/ethnicity (20, 21) but not elevated BMI (22) showed a lower immune response.

Although underlying medical conditions have been shown to be important risk factors for COVID-19 disease in adult populations (23, 24), chronic diseases are relatively rare in pediatric populations, with the exception of obesity (25). Based on the current significant gaps in our knowledge regarding the factors influencing the variability in immune reactions to SARS-CoV-2 infection, immunization, or both (hybrid) among children, this analysis aimed to (1) identify factors associated with high SARS-CoV-2 spike and nucleocapsid antibody responses; and (2) explore the distribution of nucleocapsid antibody levels by spike antibody quartiles in children and adolescents. Based on the adult literature, it was hypothesized that (1) age, sex, vaccination history, and underlying medical conditions will contribute to spike antibody response variability; and (2) hybrid immunity will demonstrate the highest SARS-CoV-2 antibody responses.

## Methods

### Study design

The Texas Coronavirus Antibody REsponse Survey (CARES) is a prospective population-based seroprevalence program designed to assess the antibody status of individuals across Texas, a large and diverse population over time. Texas CARES includes participants spanning 0 to 90 years of age from the general population, with detailed study methods previously published (2–4). We report here results only from children and adolescents ages 1-to-19 years old who had at least one antibody test and one survey completed from July 2023 to December 2023. This collaborative initiative involves the University of Texas Health Science Center at Houston School of Public Health, the Texas Department of State Health Services (DSHS), the University of Texas System, and Clinical Pathology Laboratories (CPL), a network of over 200 statewide laboratory sites. All protocols were approved by the University of Texas Health Science Center's Committee for the Protection of Human Subjects and were endorsed as public health practice by the Texas Department of State Health Services' Institutional Review Board.

### Study recruitment

Statewide enrollment for Texas CARES began in October 2020. Families of potential pediatric participants received information through diverse channels, including healthcare providers, insurance carriers (for Medicaid-insured participants), media (radio, billboards), social media, school nurses and teachers, community events, and word-of-mouth. Information was disseminated in both English and Spanish to ensure inclusivity. This recruitment strategy aimed to engage a broad spectrum of potential participants, particularly those lacking access to conventional healthcare settings or awareness of other COVID-19 research studies. Collaboration with community partners further ensured accessibility across diverse backgrounds.

### Study procedures

Interested volunteers were asked to visit a website (www.texascares.org) for more information and to participate. Parents or designated caregivers provided proxy informed consent for children and adolescents to join the Texas CARES study. Adolescents aged 12 years and above had the option to provide assent and complete the questionnaire, with none declining participation. Upon consenting to participate in Texas CARES, individuals completed a brief online questionnaire covering demographic information, employment status, baseline medical conditions, prior COVID-19 tests and diagnoses, physician-diagnosed COVID-19, height, weight, and other chronic illnesses. Following questionnaire completion, participants received orders to visit a CPL facility of their choice for the antibody status blood draw, typically receiving results within 48 h. The study team prioritized safety and convenience, allowing participants to select a laboratory facility convenient for them and providing information in both English and Spanish about the study and antibody test results.

### Study measures

#### Primary outcome variable

Spike, or S, antibody response by quartile was considered the primary outcome variable of interest in the current analysis, while nucleocapsid, or N, antibody serostatus was also evaluated. The Roche Elecsys® Anti-SARS-CoV-2 Immunoassay (Roche N-test) was used to assess SARS-CoV-2 antibody status due to naturally acquired infection, utilizing a modified recombinant protein representing the nucleocapsid (N) antigen. This highly sensitive and specific assay, with a published sensitivity of 99.50% and specificity of 99.8%, detected high-affinity antibodies to SARS-CoV-2. A few months after the commencement of Texas CARES in 2020, the Roche S-test, an immunoassay detecting antibodies to the SARS-CoV-2 spike protein, was incorporated to monitor the combined impact of prior infection and COVID-19 vaccination. Both tests have a sensitivity and specificity exceeding 97% (26, 27).

#### Primary exposure variables

Exposure to SARS-CoV-2 via natural infection or by vaccination, or both/hybrid, as well as age, body mass index, and chronic disease status, were primary exposures of interest. An online questionnaire, implemented in REDCap (27, 28) constituted Texas CARES' electronic questionnaire that captured information on infection and vaccination status was implemented at the time of each antibody test. Designed to take 10–15 min for parents or designated caregivers as proxy respondents, the questionnaire adapted questions from the COVID-19 PhenX Toolkit (29) and the Behavioral Risk Factor Surveillance System (30) questionnaires to enhance validity and reproducibility. US Census race/ethnicity questions were also replicated (31). The questionnaire seamlessly integrated with the informed consent process to streamline the respondent's experience and maximize survey completion.

Infection status was self-reported via diagnosis by a doctor, positive PCR test, or positive N-antibody test after symptoms and close contact with a positive case. Hospitalization and vaccination status were self-reported.

Weight categories were established based on calculated BMI using Centers for Disease Control and Prevention (CDC) age- and sex-adjusted BMI standardized values, categorizing participants into underweight (<5th percentile), healthy weight (≥5th to <85th percentile), overweight (≥85th to <95th percentile), and obesity (≥95th percentile) (32, 33).

## Statistical analysis

Quartile analysis divided the main dataset into four equal groups by spike antibody levels and was used for all subsequent analyses. Specifically, the quartiles divide the main dataset into four intervals, each representing 25% of the total data. The first quartile corresponds to the 25th percentile, the second quartile represents the median and corresponds to the 50th percentile, and the third quartile corresponds to the 75th percentile of S antibody levels. Quartile analysis is particularly useful in understanding the spread of data and identifying potential skewness or outliers in a more granular manner compared to traditional summary statistics. Once quartiles were established, statistical approaches included Kruskal-Wallis rank sum tests, Pearson's Chi-squared tests, and Fisher's Exact Tests to compare pediatric participant characteristics (nucleocapsid antibody status, age at the test date, BMI, days since last reported infection or vaccine, reported infection status, and vaccination status) by S antibody quartile assignment. Odds ratios (OR) and 95% confidence intervals (CI) were calculated to examine if age at the test date, days since the last known immunity challenge (in tens), and vaccination status categories (Unvaccinated, Primary Series/Partially Vaccinated, One Booster, Multiple Boosters) were significant predictors of fourth quartile membership via logistic regression analyses. The statistical significance of all associations was determined using *p*-values, with a significance level set at 0.05. All statistical analyses were performed using RStudio.

## Results

Table 1 presents the distribution of SARS-CoV-2 spike antibody quartiles among TX Cares pediatric participants (*N* = 411, mean age at test date 12.2 years, 50.6% female). Among the aggregate sample, spike values ranged from a low of 6.3 U/ml in the first quartile to a maximum of 203,132.0 U/ml in the fourth quartile. Nucleocapsid antibody mean (SD) levels were lower overall in the highest quartile vs. the third, second and first (86.2 (83.6), 98.0 (92.5), 98.3 (93.1), and 68.7 (77.6) U/ml, respectively) as well as median levels (52.2, 72.1, 76.8, and 28.4 U/ml, respectively but these differences were not statistically significant. The first quartile (*N* = 103) had a minimum spike-value of 6.3 U/ml and a maximum of 2,633.0 U/ml, while the fourth quartile (*N* = 103) ranged from 22,730.0 U/ml to 203,132.0 U/ml. Spike antibody level was significantly associated with age at the test date (*p* < 0.001), with the mean spike-value increasing with age. Similarly, categorical age analysis revealed a significant difference across age groups, with the highest quartile more prevalent in the 15–19 years old group (50.2%, *p* = 0.004). In general, participants with longer durations since last reported infection (mean 626.6 days) tended to fall in first quartile compared to those in the second (mean 439.5 days), third (mean 431.0 days) and fourth (mean 465.5 days) quartiles, respectively, *p* = 0.034). As expected, similar patterns were observed for days since last vaccine (*p* = 0.001) and days since last immunity challenge (*p* < 0.001). Similarly, vaccination status showed a trend of higher quartiles corresponding to increased vaccination: the first quartile comprised of 86.4% who were unvaccinated, while the fourth quartile had 95% who had at least one vaccine (*p* < 0.001).

**Table 1:** SARS-CoV-2 spike (S) antibody quartiles by pediatric participant characteristics, Texas CARES 2020–2023 (*N* = 411).

** **	First quartile (*N* = 103)	Second quartile (*N* = 103)	Third quartile (*N* = 102)	Fourth quartile (*N* = 103)	Total (*N* = 411)	*P*-value
S-value, U/ml						
Minimum	6.3	2,659.0	8,856.0	22,730.0	6.3	
Maximum	2,633.0	8,830.0	22,273.0	203,132.0	203,132.0	
N AB positive, *n* (%)						
Negative - no reported infection	6 (5.8)	16 (15.5)	12 (11.8)	10 (9.7)	44 (10.7)	
Negative - reported infection	1 (1.0)	2 (1.9)	0 (0.0)	3 (2.9)	6 (1.5)	
Positive - no reported infection	45 (43.7)	32 (31.1)	29 (28.4)	40 (38.8)	146 (35.5)	
Positive - reported infection	51 (49.5)	53 (51.5)	61 (59.8)	50 (48.5)	215 (52.3)	
*N*-value, U/ml						0.137^a^
Mean (SD)	86.2 (83.6)	98.0 (92.5)	98.3 (93.1)	68.7 (77.6)	87.8 (87.4)	
Median	52.2	72.1	76.8	28.4	56.0	
Range	0.0–297.0	0.0–292.0	0.0–306.0	0.0–243.0	0.0–306.0	
Age at test date, years						<0.001^a^
Mean (SD)	11.2 (4.7)	11.4 (4.1)	12.6 (3.5)	13.7 (3.1)	12.2 (4.0)	
Median	11.0	10.0	12.0	15.0	12.0	
Range	1.0–19.0	1.0–19.0	3.0–19.0	6.0–19.0	1.0–19.0	
Age at test date, categorical						0.004^b^*
Less than 5 years old	10 (9.7)	5 (4.9)	1 (1.0)	0 (0.0)	16 (3.9)	
5–9 years old	29 (28.2)	32 (31.1)	19 (18.6)	15 (14.6)	95 (23.1)	
10–14 years old	34 (33.0)	38 (36.9)	45 (44.1)	36 (35.0)	153 (37.2)	
15–19 years old	30 (29.1)	28 (27.2)	37 (36.3)	52 (50.5)	147 (35.8)	
Sex, *n* (%)						0.386^b^
Female	45 (43.7%)	54 (52.4%)	52 (51.0%)	57 (55.3%)	208 (50.6%)	
Male	58 (56.3%)	49 (47.6%)	50 (49.0%)	46 (44.7%)	203 (49.4%)	
BMI, categorical						0.585^b^
Missing	2	2	0	0	4	
Underweight	13 (12.9)	13 (12.9)	15 (14.7)	16 (15.5)	57 (14.0)	
Healthy	65 (64.4)	56 (55.4)	67 (65.7)	68 (66.0)	256 (62.9)	
Overweight	12 (11.9)	16 (15.8)	13 (12.7)	10 (9.7)	51 (12.5)	
Obesity	11 (10.9)	16 (15.8)	7 (6.9)	9 (8.7)	43 (10.6)	
Chronic condition, *n* (%)						0.244^b^
No	86 (83.5)	86 (83.5)	89 (87.3)	79 (76.7)	340 (82.7)	
Yes	17 (16.5)	17 (16.5)	13 (12.7)	24 (23.3)	71 (17.3)	
Days since last reported infection						0.034^a^
Mean (SD)	626.6 (250.9)	459.5 (229.3)	383.5 (216.8)	450.7 (312.3)	481.8 (262.0)	
Median	623.0	439.5	431.0	465.5	473.0	
Range	220.0–1,081.0	3.0–919.0	3.0–812.0	7.0–939.0	3.0–1,081.0	
Days since last reported vaccine						0.001^a^
Mean (SD)	584.8 (224.7)	605.3 (175.9)	604.3 (210.5)	425.3 (309.2)	537.3 (259.4)	
Median	583.5	625.0	637.0	414.0	615.0	
Range	320.0–1,171.0	106.0–914.0	12.0–1,013.0	10.0–911.0	10.0–1,171.0	
Days since last immunity challenge, vaccine or infection						<0.001^a^
Mean (SD)	615.3 (243.0)	562.6 (210.0)	547.9 (240.1)	382.9 (297.6)	503.0 (268.7)	
Median	606.5	619.0	593.5	394.0	553.0	
Range	220.0–1,171.0	3.0–919.0	3.0–1,013.0	7.0–911.0	3.0–1,171.0	
Reported infection, *n* (%)						0.538^b^
No	51 (49.5)	48 (46.6)	41 (40.2)	50 (48.5)	190 (46.2)	
Yes	52 (50.5%	55 (53.4)	61 (59.8)	53 (51.5)	221 (53.8)	
Number of reported infections, *n* (%)						0.572^c^
0	51 (49.5)	48 (46.6)	41 (40.2)	50 (48.5)	190 (46.2)	
1	34 (33.0)	40 (38.8)	38 (37.3)	41 (39.8)	153 (37.2)	
2	15 (14.6)	11 (10.7)	20 (19.6)	11 (10.7)	57 (13.9)	
3+	3 (2.9)	4 (3.9)	3 (2.9)	1 (1.0)	11 (2.7)	
Vaccination status, *n* (%)						<0.001^c^
Unvaccinated	89 (86.4)	41 (39.8)	11 (10.8)	5 (4.9)	146 (35.5)	
Primary Series/partially vaccinated	14 (13.6)	54 (52.4)	54 (52.9)	32 (31.1)	154 (37.5)	
One booster	0 (0.0)	5 (4.9)	22 (21.6)	20 (19.4)	47 (11.4)	
Multiple boosters	0 (0.0)	3 (2.9)	15 (14.7)	46 (44.7)	64 (15.6)	

Chronic diseases include asthma = 48, cancer = 2, cardiovascular/heart disease = 5, kidney disease = 1, high blood sugar or diabetes = 1, hypertension = 1, immunocompromised = 6, and other chronic condition = 30. Some participants had multiple diseases.

* Categorical age tested without those younger than 5 years due to sample size.

^a^ Kruskal-Wallis rank sum test.

^b^ Pearson's Chi-squared test.

^c^ Fisher's exact test with simulated *p*-value (based on 2,000 replicates).

Table 2 presents spike antibody quartiles among participants who reported at least one infection only. Overall results were similar to the aggregate sample findings; unvaccinated participants (38.5%) exhibit lower spike antibody levels compared to those with varying degrees of vaccination, including primary series/partially vaccinated (31.7%), one booster (13.1%), and multiple boosters (16.7%) (*p* < 0.001). Additionally, a significant association was shown between spike antibody quartile and the duration since the last reported infection, with decreasing mean values from 626.6 days (first quartile) to 382.9 days (fourth quartile) (*p* = 0.034). Age, place of care, and the number of reported infections, showed no significant associations with spike antibody quartiles.

**Table 2:** SARS-CoV-2 spike (S) antibody quartiles by pediatric participant characteristics with at least one infection, Texas CARES 2020–2023.

** **	First quartile (*N* = 52)	Second quartile (*N* = 55)	Third quartile (*N* = 61)	Fourth quartile (*N* = 53)	Total (*N* = 221)	*P*-value
S-value, U/ml						
Minimum	19.8	2,686.0	8,856.0	23,905.0	19.8	
Maximum	2,631.0	8,519.0	22,273.0	144,361.0	144,361.0	
Age at test date, years						0.438^a^
Mean (SD)	12.1 (4.5)	12.4 (4.0)	12.6 (3.8)	13.5 (3.0)	12.6 (3.9)	
Median	12.5	13.0	13.0	14.0	13.0	
Range	3.0–19.0	2.0–19.0	3.0–19.0	7.0–18.0	2.0–19.0	
Age at test date, categorical						0.463^b^*
Less than 5 years old	2 (3.8)	2 (3.6)	1 (1.6)	0 (0.0)	5 (2.3)	
5–9 years old	17 (32.7)	14 (25.5)	14 (23.0)	8 (15.1)	53 (24.0)	
10–14 years old	14 (26.9)	20 (36.4)	22 (36.1)	21 (39.6)	77 (34.8)	
15–19 years old	19 (36.5)	19 (34.5)	24 (39.3)	24 (45.3)	86 (38.9)	
Sex, *n* (%)						0.570^b^
Female	26 (50.0%)	27 (49.1%)	35 (57.4%)	32 (60.4%)	120 (54.3%)	
Male	26 (50.0%)	28 (50.9%)	26 (42.6%)	21 (39.6%)	101 (45.7%)	
Days since last reported infection						0.034^a^
Mean (SD)	626.6 (250.9)	459.5 (229.3)	383.5 (216.8)	450.7 (312.3)	481.8 (262.0)	
Median	623.0	439.5	431.0	465.5	473.0	
Range	220.0–1,081.0	3.0–919.0	3.0–812.0	7.0–939.0	3.0–1,081.0	
Place of care						
Hospital	0 (0.0%)	0 (0.0%)	0 (0.0%)	1 (1.9%)	1 (0.5%)	
ER	0 (0.0%)	0 (0.0%)	0 (0.0%)	0 (0.0%)	0 (0.0%)	
Doctor's office	1 (1.9%)	1 (1.8%)	0 (0.0%)	1 (1.9%)	3 (1.4%)	
At home	51 (98.1%)	51 (92.7%)	59 (96.7%)	49 (92.5%)	210 (95.0%)	
Missing	0 (0.0%)	3 (5.5%)	2 (3.3%)	2 (3.8%)	7 (3.2%)	
Number of reported infections						0.501^c^
1	34 (65.4)	40 (72.7)	38 (62.3)	41 (77.4)	153 (69.2)	
2	15 (28.8)	11 (20.0)	20 (32.8)	11 (20.8)	57 (25.8)	
3 +	3 (5.8)	4 (7.3)	3 (4.9)	1 (1.9)	11 (5.0)	
Vaccination status						<0.001^c^
Unvaccinated	48 (92.3)	27 (49.1)	9 (14.8)	1 (1.9)	85 (38.5)	
Primary series/partially vaccinated	4 (7.7)	23 (41.8)	29 (47.5)	14 (26.4)	70 (31.7)	
One booster	0 (0.0)	3 (5.5)	14 (23.0)	12 (22.6)	29 (13.1)	
Multiple boosters	0 (0.0)	2 (3.6)	9 (14.8)	26 (49.1)	37 (16.7)	

* Categorical age tested without those younger than 5 years due to sample size.

^a^ Kruskal-Wallis rank sum test.

^b^ Pearson's Chi-squared test.

^c^ Fisher's exact test with simulated *p*-value (based on 2,000 replicates).

Among those who reported no infections, the nucleocapsid-values exhibit considerable variability across quartiles, with mean positive values of 45 (88.2), 32 (66.7), 29 (70.7), and 40 (80.0) for the first through fourth quartiles, respectively (*p* = 0.049) (Table 3).

**Table 3:** *N* antibody characteristics among those reporting no infections by S antibody quartile, Texas CARES pediatric population.

** **	First quartile (*N* = 51)	Second quartile (*N* = 48)	Third quartile (*N* = 41)	Fourth quartile (*N* = 50)	Total (*N* = 190)	*P*-value
*N* antibody status						0.049^a^
Negative	6 (11.8)	16 (33.3)	12 (29.3)	10 (20.0)	44 (23.2)	
Positive	45 (88.2)	32 (66.7)	29 (70.7)	40 (80.0)	146 (76.8)	

^a^ Fisher's exact test.

Age at the test date was associated with higher odds (OR = 1.22, 95% CI: 1.12, 1.35, *p* < .001) of being in the fourth spike antibody quartile, whereas days since the last immunity challenge, represented in tens, was negatively associated (OR = 0.98, 95% CI: 0.96, 0.99, *p* = 0.002). Vaccination status showed significant associations, with primary series/partially vaccinated (OR = 13.62, 95% CI: 2.42, 261.49, *p* = 0.016), one booster (OR = 21.33, 95% CI: 3.71, 409.32, *p* = 0.005), and multiple boosters (OR = 40.77, 95% CI: 7.11, 778.11, *p* < .001) showing substantially higher odds of being in the fourth spike antibody quartile compared to those who were not vaccinated (Table 4).

**Table 4:** Odds ratios and 95% confidence intervals of fourth quartile S values, Texas CARES pediatric participants.

Variable	OR 95% confidence interval	*P* value
Age at test date	1.22 (1.12, 1.35)	<.001
Days since last immunity challenge (in tens)	0.98 (0.96, 0.99)	0.002
Vaccination status		
Unvaccinated	REF	–
Primary series/partially vaccinated	13.62 (2.42, 261.49)	0.016
One booster	21.33 (3.71, 409.32)	0.005
Multiple boosters	40.77 (7.11, 778.11)	<.001

**Figure 1:**
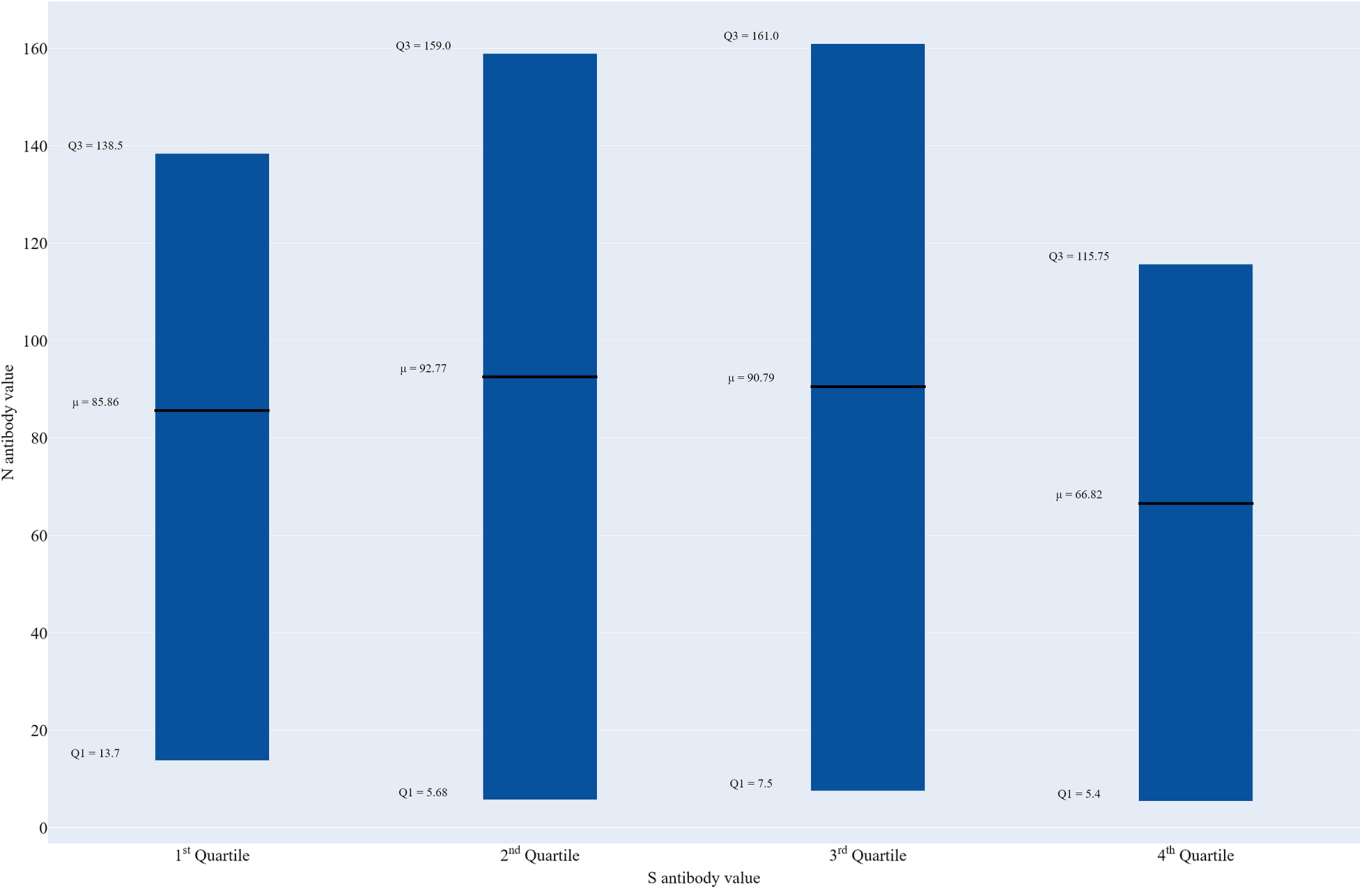
Box plot of nucleocapsid antibody levels by spike antibody quartile, Texas CARES pediatric participants (*n* = 411).

Figure 1 shows the variability in mean nucleocapsid antibody levels by spike antibody quartile. Mean N antibody levels were almost the same for the second (98.0 U/ml) and third (98.3 U/ml) quartiles, and slightly lower in the first quartile (86.2 U/ml) and lowest in the fourth quartile (68.7 U/ml), but these differences were not significant.

## Discussion

The findings from this study provide a comprehensive overview of SARS-CoV-2 spike (S) antibody responses among TX Cares pediatric participants with several key findings. First, spike antibody levels varied widely across quartiles, ranging from a low value of 6.3 U/ml in the first quartile to a high of well over 200,000 U/ml in the fourth quartile. All pediatric participants showed evidence of some spike antibodies but there were children represented with no nucleocapsid antibodies in all four quartiles. Second, vaccine and infection status, and days since last immunity challenge, were key drivers in elevated immune response in children and adolescents. And third, the age at antibody test date emerged as a significant factor, with the highest spike antibody quartile more prevalent in the 15-to-19 years old (oldest) age group vs. younger ages. It should also be noted that anywhere from 28.4% in the third quartile to 43.7% in the first spike antibody quartile had positive N antibodies but reported no infection reflecting that roughly one out of every three COVID-19 infections in children and adolescents are asymptomatic. Finally, while it was found that the highest spike antibody quartile also had the lowest mean (68.7 vs. 98.3, 98.0 and 86.2 U/ml, respectively) and median (28.4 vs. 76.8, 72.1, and 52.2 U/ml, respectively) nucleocapsid antibody levels vs. the third, second and first quartiles, respectively none of the tested differences (overall and pairwise) were significant. Overall, these findings highlight the diverse spike and nucleocapsid antibody characteristics in pediatric participants both with and without natural infection(s) and vaccinations, align with recent findings on the robust mRNA vaccine responses in children (11). However, they also raise intriguing questions about why naturally-induced nucleocapsid antibody levels may be lowest among those with the highest spike values.

Variability in immune responses, evidenced by widely differing S-values across quartiles, highlights the complexity of the pediatric immune response (12). As expected, associations between spike antibody levels and the duration since the last reported infection, vaccine, or immunity challenge were found. Specifically, longer duration since last immunity challenge was associated with lower quartiles, underscoring the impact of time on antibody duration. This finding is in agreement with other studies (3), that show robust durability of nucleocapsid and spike antibodies in a large pediatric sample up to 12 months post-infection/vaccination (1–3). However, these studies, including a systematic review of 24 seroprevalence papers (1) were from the pre-Omicron and Omicron eras. To our knowledge, this is one of the first, if not the only analysis currently available in the literature that includes the new, and dominant, JN.1. variant as of January 5, 2024.

Vaccination status independently played a pivotal role in driving the highest spike antibody response, with a clear shift towards higher quartiles corresponding to increased vaccine doses. These findings were expected, as those with more complete vaccination series, including boosters, showed substantially higher odds of being in the fourth spike antibody quartile. These findings are consistent with the literature, and crucial for understanding immune response longevity as it pertains to pediatric populations (13, 34). It should also be noted that 35.5% of the aggregate sample remained unvaccinated.

Another key finding was the significant association of older age and higher spike antibody levels. The mean age increased from 11.2 years in the first quartile to 13.7 years in the fourth quartile, demonstrating a clear age-dependent trend in spike antibody responses. Categorically, the prevalence of the highest quartile was notably higher in the 15-to-19 years old group (50.5%), compared to the other age categories. These findings may be the result of vaccination only being available to older children and adolescents early in the pandemic and hesitancy of parents to vaccinate very young children. These findings are consistent with other literature demonstrating that age is positively correlated with spike protein antibody response among children and young adults (13).

Our results did not show any significant associations between BMI group and chronic conditions with S antibody quartiles. These finding are noteworthy, as it suggests that the immune responses to SARS-CoV-2 in pediatric individuals may not be significantly compromised by the presence of chronic health conditions, including obesity in the same way that it might be in certain adult populations. Indeed, adult studies show that comorbidities may enhance humoral response to SARS-CoV-2 due to increased pro-inflammatory states (14) while other studies show the presence of chronic conditions (autoimmune disorders, diabetes and kidney disease) (18) showed lower immune response.

Results showed that a substantial proportion (28.4%–43.7% across quartiles) of participants with positive N antibodies reported no COVID-19 infection(s). Conversely, only 1.0%–2.9% with negative N antibodies reported a previous infection. These results suggest that a significant number of children and adolescents continue to experience asymptomatic, and thus unreported COVID-19 infections, which has been an important hallmark of the COVID-19 pandemic since its inception (35).

Collectively, these findings are informative for pandemic science and public health. Specifically, findings here underscore the importance of understanding pediatric immune responses for developing effective vaccination strategies and managing reinfection risks and long-term health outcomes. Trends in both spike and nucleocapsid antibody levels, age-dependence, and vaccination status form a basis for targeted health interventions, especially in pediatric healthcare and vaccination efforts.

### Study limitations and strengths

This study is not without limitations, notably, the reliance on self-reported data, which may introduce recall bias. Second, all COVID-19 disease and vaccination data were self-reported. Third, Texas CARES participants may be different in terms of awareness, willingness, and ability to participate vs. the overall state population on important social determinants of health (e.g., education, socioeconomic level, language other than English or Spanish), resulting in selection bias. Despite this, Texas CARES offers valuable insights into both the spike and nucleocapsid antibody response to SARS-CoV-2, supported by its robust recruitment strategy, sensitive antibody assays from Roche Diagnostics, and a comprehensive electronic questionnaire.

## Conclusion

In summary, this study identified factors associated with heightened antibody responses in children and adolescents to identify potential unique immune dynamics in this population. Findings highlight considerable variability in S antibody levels across quartiles, with intriguing patterns in the relationship between S and N antibody levels. Older age, less days since last immunity challenge and vaccination status were associated with heightened S antibody immune response in children and adolescents. Notably, a significant percentage of participants with positive N antibodies reported no infection, underscoring the continued prevalence of asymptomatic cases in pediatric COVID-19 infections. Overall, these results contribute to our understanding of antibody characteristics in pediatric populations, aligning with broader trends observed in recent studies on vaccine responses in children.

## Data Availability

Texas CARES investigators are committed to data sharing. Granular results and user-specified data summaries are currently publicly available on the Texas CARES portal (https://sph.uth.edu/projects/texascares/dashboard). After project completion, de-identified secondary data and detailed documentation will be made publicly available through the same portal as open-access data sources after de-identification and collapsing cells to protect individuals representing rare events or conditions. De-identified datasets will be made available upon reasonable request and within a reasonable time in accordance with the general spirit of colleagueship within the scientific community and with policies adopted by UTHealth Houston and the Texas Department of State Health Services. Please submit data requests to institutionalreviewboard@dshs.texas.gov.
